# Supplementary material for: Photonic Stochastic Emergent Storage for deep classification by scattering-intrinsic patterns
Source: Nat Commun. 2024 Jan 13;15:505. doi: 10.1038/s41467-023-44498-z (PMC10787794; doi:10.1038/s41467-023-44498-z)
Supplement: Supplementary file 1 — Supplementary informations [file 41467_2023_44498_MOESM1_ESM.pdf]

Supplementary Informations: Photonic Stochastic Emergent Storage for deep classification by scattering-intrinsic patterns

Marco Leonetti,[1,2,3,*] Giorgio Gosti,[1,2] and Giancarlo Ruocco[2,4]

[1] *Soft and Living Matter Laboratory, Institute of Nanotechnology, Consiglio Nazionale delle Ricerche, 00185 Rome, Italy*
[2] *Center for Life Nano- & Neuro-Science, Italian Institute of Technology, Rome,Italy*
[3] *Rebel Dynamics-IIT CLN2S Jointlab, 00161 Roma Italy*
[4] *Department of Physics, University Sapienza, I-00185 Roma, Italy*

(Dated: December 13, 2023)

Full-Hardware SES and Scalability

SES can be implemented in two distinct versions: partially hardware-based and fully hardware-based. In the partially hardware-based version, as demonstrated earlier, the processes of pixel/mode selection and neutral density filtering to achieve the λ^ν coefficients are achieved through software-based multiplication, wherein individual intensity values I^ν are modulated by relative absorption coefficients.

In the fully hardware-based version, absorption coefficients can be realized using hardware masks that either fully absorb light for irrelevant modes or attenuate light by a factor of λ^ν through neutral density filtering for selected M^* modes. However, it's important to note that the fabrication of such masks can be challenging due to the requirement for micron-level precision during fabrication and fine alignment during assembly. These obstacles can be effectively overcome through the implementation of an adaptive optics layer.

This alternative approach, relies on a second Digital Micromirror Device (DMD), serving as a programmable and reconfigurable absorptive mask, referred to as a "mode selector" DMD. With this approach, a single optical setup can be employed to store multiple memories, eliminating the need for the individual fabrication of attenuation masks for each memory. When testing or probing against a memory, it becomes a matter of rearranging the mode selector DMD, rather than creating an entirely new optical setup with mode-specific absorptive masks. This streamlined approach enhances the flexibility and scalability of the SES system, making it more adaptable to various memory storage and retrieval tasks.

To achieve the required 4 bit depth required in the $\lambda^n u$ values, the mode selection DMD is organized into 4×4 superpixel, (so that each superpixel corresponds to a mode ν) which are turned on with a specific ratio of ON/OFF pixels to realize the required absorption. Eventually a second iris can be added after the mode selection DMD, in order to blur together, the grainy structure. This DMD based approach, not only enables a fabrication free approach, but also avoids the need for fine alignment, as the alignment can be performed, *ex-post* by mapping DMD superpixels on measured modes. Also this DMD-based approach, enables higher flexibility, so that a single probe pattern, can be "tested" against different memories at kHz speed.

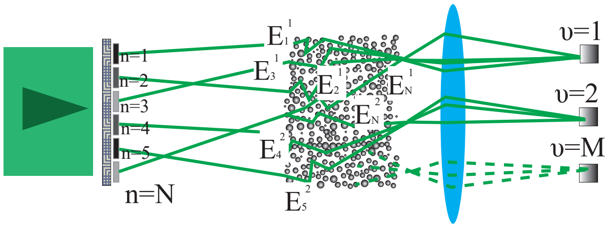

Supplementary Figure 1. Sketch of the experimental setup employed in the main paper including naming for the input mode/segments (n index) and detectors/output modes ν index

By employing a commercially available DMD with 4 million pixels , 250 thousand modes can be easily probed at 10 kHz speed. This approach is also scalable: our experiment Has been realized with a 0.5 Watts CW power laser while we tested transmission matrix stability up to 20 Watts illumination. Thus considering incremental losses, the scheme can be replicated on at least 10 times without any fundamental obstacle other than optical complexity. A sketch of the experimental scheme is provided in the Supplementary Figure 2 above. As an example an hardware configuration with the performances fulfilling the requirements is: *i)* the DLP9000X texas instumrent DMD and related DLPC910 controller for both probe and selection DMS; *ii)* FDS100 from thorlabs as large area photodyode, and *iii)* Basler a2A2448-75umPRO camera for mode characterization .

Data compression with SES

Storing a memory with SES has a twofold advantage. First, since the memory is written in an optical layer, it is ready for further operations that may be performed at the speed of light, such as for example classification via deep-SES. Second, SES written memories provide a form of (lossy) data compression. To retrieve a memory we require the address of the pixels composing M^* selected modes (16 bits for a $M^L = 65536$ repository),

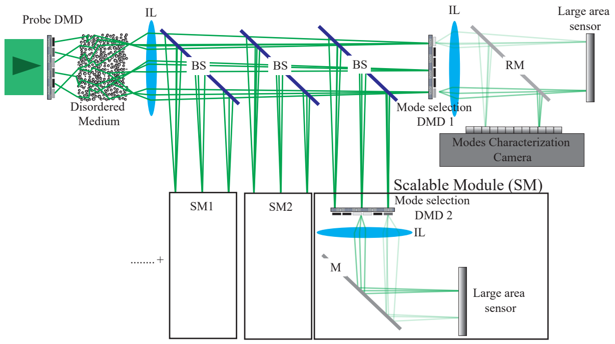

Supplementary Figure 2. Sketch for re-configurable, and scalable SES. Optical Elements acronyms : IL: imaging Lens; BS: Beam Splitter; RM: Removable Mirror; M: Mirror. The probe pattern $\boldsymbol{\phi}$ is injected in the disordered classifier by the probe DMD. Light at the back focal plane of the disordered medium is imaged on a Mode selection DMD, which transmits selected modes and introduces the weights $\lambda^n u$. A camera is present for the optical synaptic matrix, Transmission matrix measurement, while at the classification, memory retrieval stage the RM mirror is removed and the *transformed intensity* is retrieved by a single large area detector to increase the measurement speed. In the "Pattern probing" configuration, a single memory is "printed" on the Mode selection DMD while multiple probe patterns are cycled and *transformed intensity* is retrieved for each pixel thus retrieving the degree of similarity of a single memory against many probe patterns and retrieving the most pattern most similar to the chosen memory. On the contrary, a single probe pattern can be tested against many memories, by cycling on the Mode Selection DMD (like in Figure 4 a of the main paper.). Note that the scalable Module part can be replicated enabling to test a single pattern simultaneously against many different memories in parallel.

and the values of the lambdas (4 bits) for the attenuation. This is a total of $20M^*$ bits per memory. For a pattern requiring N bits to be stored, this means in the regime $20M^* < N$ we obtain an effective data compression. Obviously, this data compression comes at a cost because SES storage comes with a certain error percentage (see figure 3a in the main paper). In Supplementary Figure 3, we report the compression performance versus M^* for an $N = 256$ pattern. In the green shaded area, there is a 1/(compression ratio) smaller than unity thus highlighting the efficient compression regime.

Pattern recognition sketch

The Supplementary Figure 4 provides a visualization for the classification/recognition process.

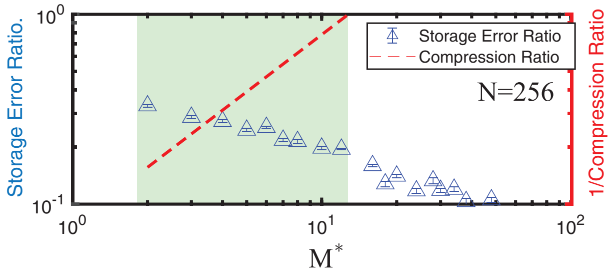

Supplementary Figure 3. Compression performance for a $N = 256$ (dashed line). The green shaded area is the area with efficient compression. Also the retrieval error is reported (blue triangles).

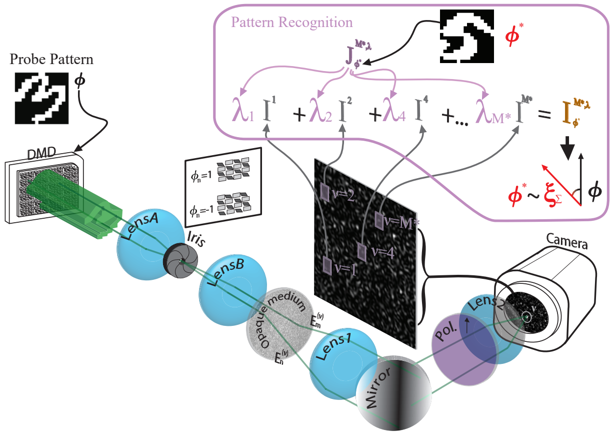

Supplementary Figure 4. Sketch of the pattern recognition and classification. The task is to assess which pattern into a set of probe patterns corresponds to one specific pattern memorized into the disordered classifier. This stage is carried on after the memory writing process has already been finalized, then the set of most relevant pixel/modes and the specific weights lambdas are already known. Then the first probe pattern is presented to the disordered classifier trough the DMD. Intensity from the selected M^* pixels is retrieved and summed with digital operation or analogically. The retrieved transformed intensity, provides an analogical measure of the degree of similitude between the probe and the stored pattern ϕ^*. This process is then repeated for all the patterns in the set of of probe patterns. The recognized pattern is the pattern producing the higher intensity.

Comparison with other optical platforms

We have conducted a comprehensive comparison of our results with other architectures that rely on free-space transformations, specifically Diffractive Deep Neural Networks ($\mathrm{D^2NN}$) [1] and Ridge Regression With Speckles (RRS) [2].

$\mathrm{D^2NN}$, as reported in [1], is based on the design of transmission matrices achieved through iterative heavy-weight computation of optical propagation. This process involves multiple optical simulations, and the resulting node weights are subsequently translated into physically

printed scattering layers for optical experiments. Notably, the creation of the artificial disordered structure in D^{2}NN demands substantial computational effort and a subsequent fabrication stage, both of which are unnecessary in both SES/Deep-SES and RRS.

RRS instead requires strong computational effort (inversion of a big size matrix) and an high number of detectors (large M^*).

In Supplementary Table I, the second column outlines the capability to read and write optical memories. With SES, we have demonstrated the capability for both writing and reading (W/R) memories, along with the ability to store up to 4096 memory elements without any fabrication effort. In contrast, RRS does not possess memory storage capabilities. Although DDNN inherently provides the possibility to write hardware memories (even though this aspect may not be explicitly detailed in the paper), it's worth noting that there is an intrinsic advantage in having a memory instantiated within the optical layers, as these layers are readily amenable to further optical operations.

The third column of Supplementary Table I outlines the classification performance for $M^* = 120$. It's important to note that in both SES and RRS, M^* represents the number of camera pixels employed, thus directly influencing the hardware resource requirements and overall protocol complexity. Additionally, it's worth highlighting that DDNN operates on a different paradigm, where M^* does not serve as the relevant parameter for evaluating computational-experimental complexity.

The fourth column of Supplementary Table I reports on the ability to obtain analog output. Both DDNN and SES produce outputs in the form of optical intensity, making them compatible with subsequent analog operations, potentially enabling faster processing. The paper on RRS [2] does not provide information on this capability.

In SES, the 'transformed intensity' corresponding to each memory serves as an analogical measure of the probe's degree of similarity with the stored memory. This unique feature enables the realization of Content Addressable Memory (CAM), which not only provides the address of the most relevant retrieved memory but also quantifies the degree of similarity with other stored memories. It's important to note that this special capability is unattainable in the other two platforms, as documented in the fifth column of Supplementary Table I.

Geometrical Interpretation

In SHS and in SES, given a pattern ϕ^* and its respective synaptic matrix $\mathbf{T}$ we want to derive a weighted sum of M matrices $\mathbf{V}^\nu$ that optimizes a certain cost function. In SHS the cost function is given by Eq. (29) and in SES it is given by Eq. (37). From the observation that $\mathbf{V}^\nu = \boldsymbol{\xi}^\nu \otimes \boldsymbol{\xi}^{\nu\dagger} + \boldsymbol{\eta}^\nu \otimes \boldsymbol{\eta}^{\nu\dagger}$, we know that each $\mathbf{V}^\nu$ has only two non-negligible eigenvalues-eigenvectors couples. If $M < N$, and if we assume that ϕ^*, ξ^ν, and η^ν are statistically independent N-dimensional vectors, we can assume that the collection of these vectors forms a $2M+1$ dimensional basis, $Q = \{\phi^*, \xi^0, \xi^1, \ldots, \xi^M, \eta^0, \eta^1, \ldots, \eta^M\}$. If we use Gram–Schmidt process we can derive an orthonormal basis P corresponding to Q, such that $P = \{\phi^*, v^0, v^1, \ldots, v^K\}$, where $K = \min(2 * M, N-1)$. It follows that we can write the synaptic matrix $\mathbf{T}$ as:

$$\mathbf{T} = P \Lambda_{\phi^*} P^T \tag{1}$$

where

$$\Lambda_{\phi^*} = \begin{bmatrix} 1 & 0 & \ldots & 0 \\ 0 & 0 & \ldots & 0 \\ \vdots & \vdots & \ddots & \vdots \\ 0 & 0 & \ldots & 0 \end{bmatrix}$$

Similarly, we can write

$$\begin{aligned} \mathbf{J}_{\boldsymbol{T}}^{\boldsymbol{\mathcal{M}},\boldsymbol{\lambda}} &= \sum_{\nu}^{M} \lambda_\nu V^\nu = \sum_{\nu}^{M} \lambda_\nu (\xi^\nu \otimes \xi^{\nu\dagger} + \eta^\nu \otimes \eta^{\nu\dagger}) \\ &= \sum_{\nu}^{M} \lambda_\nu P A^\nu P^T \end{aligned} \tag{2}$$

where A^ν is a $N \times N$ matrix

$$A^\nu = P^T (\xi^\nu \otimes \xi^{\nu\dagger} + \eta^\nu \otimes \eta^{\nu\dagger}) P$$

Consequently, in SHS, optimizing Eq. (29) is equivalent to optimizing $\mathcal{F}(\boldsymbol{\mathcal{M}}, \boldsymbol{\lambda}) = \sum (\Lambda_{\phi^*} - \sum_\nu \lambda_\nu A^\nu)^2$. Clearly, as M tends to N we get to a perfect reconstruction of $\mathbf{T}$. In SES, the interpretation is a little less straightforward because we are optimizing the projection of ϕ^* with the largest eigenvector of $\mathbf{J}_{\boldsymbol{\phi^*}}^{\boldsymbol{\mathcal{M}}^*,\boldsymbol{\lambda}}$. What happens is that we tend to constructively sum the weighted projections of V^ν parallel to ϕ. Meanwhile, we can assume that the perpendicular terms of V^ν are randomly weighted. Thus the contribution of the perpendicular terms of V^ν vanishes for M growing to infinite, but it is not negligible for finite intermediate values of M. In conclusion. while in SHS we optimize simultaneously the parallel and perpendicular contribution of V^ν, in SES we optimize only the parallel contribution of V^ν. This is the reason why in Figure 2 and Figure 3 of the main paper, we see that given a random subset $\boldsymbol{\mathcal{M}}$ with M modes SHS performs better than SES. Nevertheless, the contribution of the perpendicular terms of V^ν is strongly reduced when we select the V^ν with the strongest values of similitude degree $\mathcal{S}^\nu = \hat{\boldsymbol{\phi}}^* \cdot \hat{\boldsymbol{\xi}}^\nu$, because we are selecting the V^ν with the smaller perpendicular component. This is why in Figure 3 of the main paper we see a clear difference between using the random $\boldsymbol{\mathcal{M}}$ or the sorted $\boldsymbol{\mathcal{M}}^*$.

Supplementary Table I. Comparison Between SES, Deep-SES, RRS[2], DDNN[1] . Second column repots about the Write capability(W) and Read capability (R). The third column report efficiency is (defined as the ratio of the number of elements appearing in the diagonal in the confusion matrix with respect to all the elements of the same matrix). Note that for DDNN (efficiency appearing with an asterisk in the third column), the dataset is different that used in SES, Deep-SES, and RRS. "Analog output" column report the possibility to gather the output in the form of intensity. The third column report the possibility of the platform to work as Content Addressable Memory, and to employ Intensity as a measure of the similarity between the stored memory and the probe pattern.

Architecture	Memory Storage	Efficiency (M^* =120)	Analog output	CAM and Similarity
SES	W/R	-	✓	✓
Deep SES	W/R	91.71 %	✓	✓
RRS[2]	none	53.54 %	✗	✗
DDNN[1]	W	91.75* %	✓	✗

* marco.leonetti@cnr.it

[1] X. Lin, Y. Rivenson, N. T. Yardimci, M. Veli, Y. Luo, M. Jarrahi, and A. Ozcan, All-optical machine learning using diffractive deep neural networks, Science **361**, 1004 (2018).

[2] A. Saade, F. Caltagirone, I. Carron, L. Daudet, A. Drémeau, S. Gigan, and F. Krzakala, Random projections through multiple optical scattering: Approximating kernels at the speed of light, in *2016 IEEE International Conference on Acoustics, Speech and Signal Processing (ICASSP)* (IEEE, 2016) pp. 6215–6219.
